# Comparing localized and nonlocalized dynamic ^31^P magnetic resonance spectroscopy in exercising muscle at 7T

**DOI:** 10.1002/mrm.24205

**Published:** 2012-02-14

**Authors:** Martin Meyerspeer, Simon Robinson, Christine I Nabuurs, Tom Scheenen, Adrian Schoisengeier, Ewald Unger, Graham J Kemp, Ewald Moser

**Affiliations:** 1 Center for Medical Physics and Biomedical Engineering, Medical University of ViennaVienna, Austria; 2 MR Centre of Excellence, Medical University of ViennaVienna, Austria; 3 LIFMET/CIBM, Ecole Polytechnique Federale de LausanneLausanne, Switzerland; 4 Department of Radiology, Medical University of ViennaVienna, Austria; 5 Hôpitaux Universitaires de GenèveGeneva, Switzerland; 6 NUTRIM School for Nutrition, Toxicology and Metabolism, Department of Human Movement Sciences & Radiology, Maastricht University Medical Centre+Maastricht, the Netherlands; 7 Department of Radiology, Radboud University Nijmegen Medical CentreNijmegen, The Netherlands; 8 Department of Musculoskeletal Biology, Magnetic Resonance & Image Analysis Research Centre, University of LiverpoolLiverpool, United Kingdom

**Keywords:** exercise, phosphocreatine, energy metabolism, ^31^P

## Abstract

By improving spatial and anatomical specificity, localized spectroscopy can enhance the power and accuracy of the quantitative analysis of cellular metabolism and bioenergetics. Localized and nonlocalized dynamic ^31^P magnetic resonance spectroscopy using a surface coil was compared during aerobic exercise and recovery of human calf muscle. For localization, a short echo time single-voxel magnetic resonance spectroscopy sequence with adiabatic refocusing (semi-LASER) was applied, enabling the quantification of phosphocreatine, inorganic phosphate, and pH value in a single muscle (medial gastrocnemius) in single shots (*T*_R_ = 6 s). All measurements were performed in a 7 T whole body scanner with a nonmagnetic ergometer. From a series of equal exercise bouts we conclude that: (a) with localization, measured phosphocreatine declines in exercise to a lower value (79 ± 7% cf. 53 ± 10%, *P* = 0.002), (b) phosphocreatine recovery shows shorter half time (*t*_1/2_ = 34 ± 7 s cf. *t*_1/2_ = 42 ± 7 s, nonsignificant) and initial postexercise phosphocreatine resynthesis rate is significantly higher (32 ± 5 mM/min cf. 17 ± 4 mM/min, *P* = 0.001) and (c) in contrast to nonlocalized ^31^P magnetic resonance spectroscopy, no splitting of the inorganic phosphate peak is observed during exercise or recovery, just an increase in line width during exercise. This confirms the absence of contaminating signals originating from weaker-exercising muscle, while an observed inorganic phosphate line broadening most probably reflects variations across fibers in a single muscle. Magn Reson Med, 2012. © 2012 Wiley Periodicals, Inc.

Phosphorus-31 NMR spectroscopy has been used over three decades to study the metabolic response of muscle tissue to exercise and recovery (1). Using ^31^P magnetic resonance spectroscopy (MRS), metabolite concentrations can be quantified as ratios or absolute values, and rate constants of high-energy phosphates can be measured noninvasively in tissues such as muscle (2–11) and brain (12, 13) either in response to a physiological stimulus or under steady-state conditions by magnetization transfer experiments.

The great majority of studies using dynamic ^31^P MRS experiments in skeletal muscle during rest, exercise, and/or ischaemia and subsequent recovery have been performed without gradient-based localization methods, i.e., the volume of interest (VOI) has been defined by the sensitivity profile of the surface coil. In such experiments, the required temporal resolution is dictated by, e.g., the time constant of postexercise PCr resynthesis, a parameter associated with oxidative capacity, which is on the order of half a minute or less. A temporal resolution on the order of seconds is, therefore, required to resolve this time course and to fit a model of, e.g., monoexponential kinetics with a sufficient number of data points in relation to the model's degrees of freedom. High time resolution is also required for calculation of parameters related to cellular H^+^ efflux and buffering from measured inorganic phosphate (Pi) and negative logarithm of hydrogen ion concentration (pH) dynamics.

Localized spectroscopy was first used to study muscle exercise response two decades ago (14, 15). In these studies, the heterogeneity of pH was investigated from data acquired after exercise or as the average over the last minutes of an exercise bout (16). Despite these earlier works on localized ^31^Pmuscle MRS, to our knowledge, the time courses of measured PCr, Pi, and pH derived from nonlocalized dynamic experiments with continuous data acquisition, as typically used for studying muscle metabolism, have not yet been compared directly to a localized spectroscopic experiment selecting a single exercising muscle, with the same temporal resolution. Instead, it has been assumed either that only the targeted muscle contributes to measured metabolic changes or that contributions from nonexercising muscle do not materially affect metabolic rates calculated or conclusions inferred from these data.

Recently, the feasibility of acquiring spatially resolved ^31^P data has been demonstrated in exercising muscle using chemical shift imaging (17) or direct imaging of PCr or both PCr and Pi (18, 19). These approaches are attractive tools to study the spatial distribution of metabolic changes but have inherent shortcomings. Chemical shift imaging methods require relatively long acquisition times. This can be accommodated by gating the exercise, but then assumptions about the shape of the metabolites' recovery time courses are implied, e.g., exponential recovery to base line and/or toward a steady-state or driven equilibrium. Additionally, the real voxel size for chemical shift imaging acquisitions can be much larger than the nominal voxel size when low matrix sizes are chosen, especially when acquisition is accelerated by, e.g., elliptical *k*-space sampling and acquisition weighting. This may lead to misinterpretations by under-estimating (or ignoring) signal contamination from outside the VOI. Imaging methods, alternatively, lack chemical shift information and hence afford no insight into intracellular pH, which is of considerable physiological relevance.

The increased specificity of localized MRS comes at the cost of lower SNR per unit time. This may necessitate temporal averaging, thereby reducing temporal resolution. At lower field strengths, a direct comparison of localized and nonlocalized dynamic muscle ^31^P NMR spectroscopy is hampered by limited SNR per unit time (in particular at 1.5 T), in combination with prolonged *T*_1_ relaxation times at 3 T (20). The increased SNR (21, 22) and shorter *T*_1_ relaxation times of ^31^P metabolites at 7 T (23) provide the technical conditions to bridge the gap between high temporal resolution, nonlocalized ^31^P MRS and dynamic, localized MRS.

Localizing the signal in dynamic ^31^P spectroscopy seems a particularly advantageous approach, when the sensitive volume of a coil covers a significant part of an exercising limb. In this case, the signal contains contributions from a heterogeneous mixture of muscle groups, potentially with different fiber compositions, which may show different responses to exercise.

The hypothesis of this work is that localizing the signal to a single exercising muscle will help overcome the lack of spatial specificity of nonlocalized data, which may otherwise lead to potentially incorrect quantitative conclusions in the interpretation of bioenergetic data. To test this hypothesis, nonlocalized pulse-acquire ^31^P MRS was compared to dynamic localized ^31^P MRS of a particular exercising muscle, the gastrocnemius medialis during plantar flexion. The localization scheme used was a gradient-based single-shot, single-voxel method using conventional slice selective excitation combined with localizing adiabatic selective refocusing (semi-LASER) (24–26) with short echo time (*T*_E_), which we described in detail in Ref.^27^. Several parameters characterizing muscle exercise and recovery are compared quantitatively.

## SUBJECTS AND METHODS

Ten healthy subjects (three females, aged (mean ± SD) 28.2 ± 7.8 y, BMI = 23.8 ± 3.0 kg/m^2^) performed plantar flexion exercise in a supine position on a custom built pneumatic pedal ergometer (28). First, the study protocol was explained, and the subjects gave written consent, in accordance with the approval given by the local ethics committee. The dominant leg of subjects, who lay supine on the patient bed, was fixed in the ergometer. Subjects were asked to perform a small number (about 5) plantar flexions to practice achieving the prescribed pace and to check for good positioning and fixation. The prescribed pace was two plantar flexions per repetition time of the sequence (6 s) over the normal range of foot flexion. The recoil force of the ergometer's pedal was adjusted via the pressure in the pneumatic system and was measured using nonmagnetic sensors in the pedal or via the pressure in the cylinder of the ergometer. Seven subjects exercised at 50% of maximal voluntary contraction force (MVC, measured on the same ergometer), two subjects at a reduced force level of 30% MVC. To minimize motion-related artifacts (i.e., localization of different fractions of muscle tissue due to the altered shape of contracting muscles, which would also cause changes of line width and coil load), subjects were trained to return their foot to the neutral position before each MR excitation and acquisition. Subjects were trained to use the gradient noise to time their exercise. (The pulse-acquire sequence comprised a spoiler gradient at the end of the sampling period.) The pedal force was adjusted via the pressure in the ergometer's pneumatic system to achieve submaximal exercise while yielding sufficient phosphocreatine (PCr) depletion without excessive intracellular pH change (see “Results” section). Before starting the exercise bouts, localizer images were acquired, the VOI was shimmed, and RF pulse power was adjusted individually for each subject. ^31^P MRS and exercise thus started approximately 45–60 min after the subjects' arrival on site, during which time they were mostly physically inactive.

**Fig 1:**
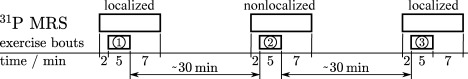
Study protocol, consisting of three equally intense plantar flexion exercise bouts with 5 min duration, spaced by 30 ± 12 min of physical inactivity. ^31^PMR spectra of human calf muscle were acquired localized, nonlocalized, and localized, with 6 s time resolution throughout.

Each subject performed three exercise bouts in series to acquire sets of localized, nonlocalized and then a second set of localized ^31^P MR spectra. Each measurement consisted of the acquisition of baseline data during 2 min of rest, 5 min of aerobic plantar flexion exercise, and 7 min of recovery, as shown in Fig. 1. The interval between the end of the exercise and the onset of the following bout was 30 ± 12 min, during which the position of the calf was checked using localizer images and the VOI was reshimmed, while the subjects stayed in place. This procedure was adopted on the one hand to capture potential variations in measured parameters caused by repeating the exercise bout, which would be apparent from the comparison of the first and third bout, and on the other hand to be able to directly compare data from the second (nonlocalized) and third (localized) exercise bout, which were hence both executed after a prior bout.

### MRS Pulse Sequences

A dual-tuned loop coil was used for RF transmission and reception of NMR signals. The coil, with a diameter of 10.5 cm for ^31^P and 9.5 cm for ^1^H (Rapid Biomedical, Würzburg, Germany), was interfaced to a Siemens 7 T whole-body MR system (Siemens Medical Solutions, Erlangen, Germany). The manufacturer's implementation of a 3D map shim was used for localized first and second-order shimming the VOI.

**Fig 2:**
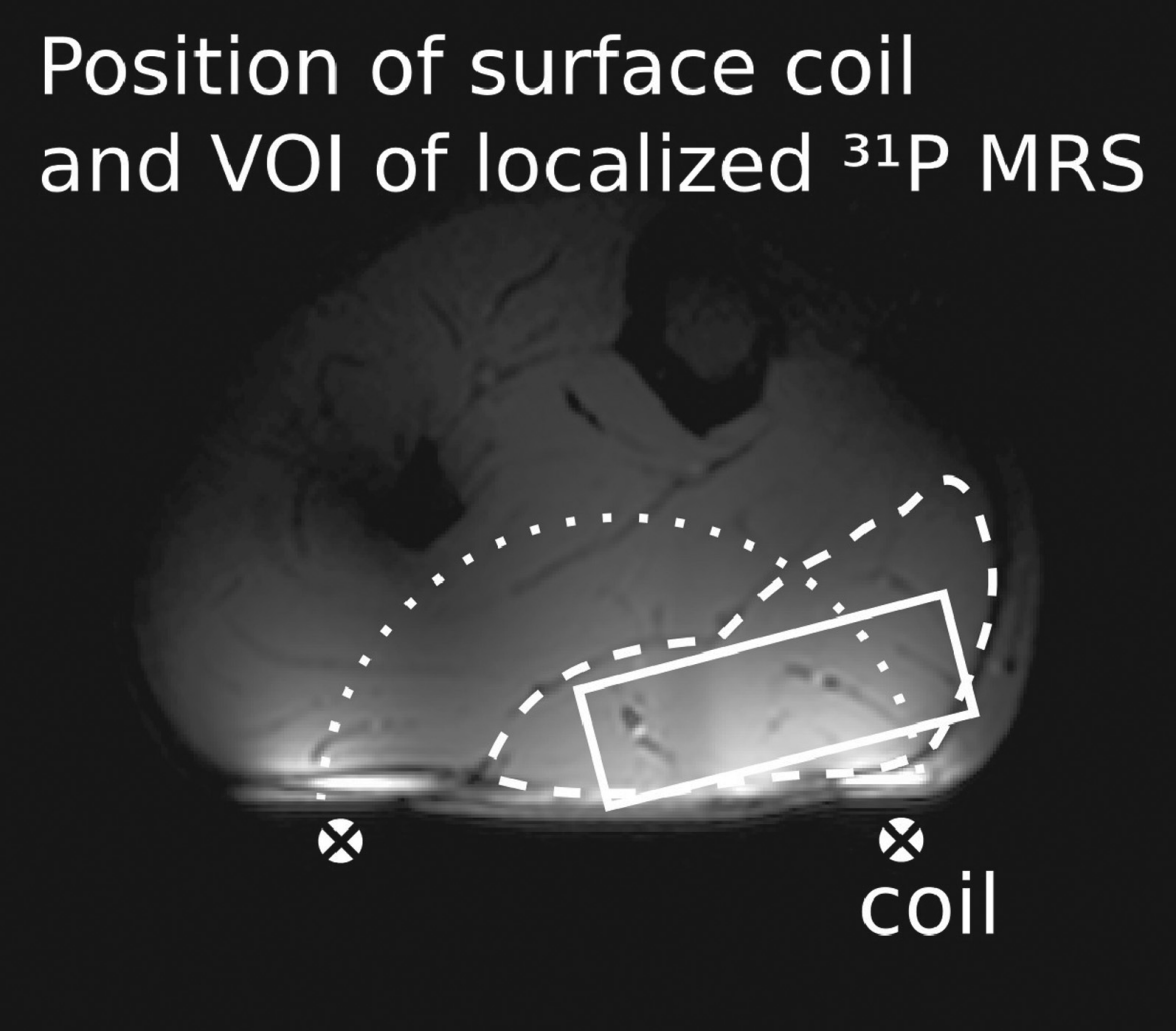
Position of the VOI (rectangle) in medial gastrocnemius muscle (dashed line). The sensitive volume of the surface coil (crosses) is indicated as a dotted half circle.

The nonlocalized measurements were performed with a pulse-acquire scheme comprising a 0.25-ms block pulse followed by the acquisition. The RF transmit voltage was adjusted for maximum signal. In all ^31^P MRS experiments, the acquisition bandwidth was 5000 Hz with 2048 complex data points, and each acquisition vector was stored separately, without averaging. For localized experiments, a double-oblique voxel was placed in the subjects' medial gastrocnemius muscle (Fig. 2), which was selected using semi-LASER, a single-shot method comprising a classic excitation pulse and two pairs of adiabatic refocusing pulses (27). The size of the VOI was adjusted so as to be constrained to the muscle, VOI dimensions ranged from 24 to 57 cm^3^, with average dimensions of 4.4 × 1.7 × 5.4 cm^3^. The VOI position was determined using scout image scans with three orthogonal slices and multislice (NS = 14) gradient echo images. Calibration of the RF transmit voltage for achieving a 90° excitation tip angle and fulfilling adiabatic conditions in the VOI was verified, individually for each subject, by varying the RF transmit voltage until a maximum of the PCr signal was reached for the given geometry.

Medial gastrocnemius is expected to be the most intensely working muscle during plantar flexion exercise with an extended knee. It consists predominantly of fast-twitch fibers, and its cross-sectional area occupies ca. 20% of the total cross-sectional area of calf muscles at the position selected for spectroscopy. The contamination of signal by contributions from outside the VOI with 1.1 ± 0.5% (27) is negligibly small with this localization scheme, and the VOI did not cover any other muscles. Therefore, we can conclude that the measured muscle signal is recorded almost exclusively from medial gastrocnemius.

The semi-LASER sequence (27) was optimized by selecting a repetition time of *T*_R_ = 6 s to achieve high SNR per unit time for the PCr and Pi resonances that have *T*_1_ relaxation times of 4.0 ± 0.2 s and 6.3 ± 1.0 s, respectively, at 7 T (23). To achieve minimum echo time, pulse durations of the adiabatic smoothed chirp pulses were adapted between 3.0 and 3.8 ms depending on required RF power. This resulted in echo times of *T*_E_ = 23 ms (seven subjects), 24 ms (one subject), and 26 ms (two subjects) and corresponding refocusing bandwidths of 7.6–6 kHz.

### Processing of MRS Data

Spectra were quantified with jMRUI (29), using the AMARES (30) time domain fit routine. Gaussian line shapes were found to result in a better match to line shapes of the localized in vivo data than Lorentzian shapes that were applied for fitting nonlocalized data. Widths of the lines are given using the relation between the damping parameter Γ/π of a gaussian line (31) and full width at half maximum according to 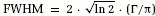. Cytosolic pH was calculated according to Ref.^32^, using the chemical shift difference δ between Pi and PCr, as pH = pK + log [(δ − δ_HA_)/(δ_A_ − δ)], with p*K* = 6.75, δ_HA_ = 3.27, and δ_A_ = 5.63. When two Pi resonances became discernible (which occurred in nonlocalized data sets only), they were fitted as two separate peaks, constraining the chemical shift to either more (Pi1) or less than (Pi2) 4.5 ppm relative to PCr, which corresponds to a pH of 6.8. No minimum separation in chemical shift of the peaks was supplied in the prior knowledge.

PCr and Pi signal intensities were quantified from single acquisitions to fit PCr recovery to an exponential model, individually for each subject, and to follow the time course of Pi, pH, and the sum of PCr and Pi as group averages using the Python programming language (http://www.python.org/). Additionally, for Pi quantification and to establish pH values during rest and recovery when Pi concentration is low, the raw data of localized spectra from single subjects were averaged along the temporal axis, to improve SNR at the cost of a reduced temporal resolution. Pi levels were assumed to be constant within the averaging window under baseline conditions as well as in the late recovery phase. The fit parameters for PCr resynthesis were the rate constant *k*, given in s^−1^, the end exercise PCr depletion *d*, expressed as a percentage of resting PCr signal, and the resting PCr signal intensity *m*, according to the function *f*(*t*) = *m* ċ[1−*d* ċ exp(−*tk*)]. In the “Results” section, the kinetics of PCr recovery is described by the half time *t*_1/2_ (in seconds), which is reciprocally proportional to the rate constant according to *t*_1/2_ = ln(2) ċ *k*^−1^.

### Intracellular Acid–Base Calculations

Initial-exercise changes in pH and PCr were used to estimate cytosolic non-Pi buffer capacity β as described in Refs.^33^ and^34^. Briefly, the net breakdown of 1 mol of PCr can be thought of as resulting in the consumption of −γ mol H^+^, where γ is a negative stoichiometric coefficient depending on the charge difference of Pi and PCr (34, 35). In early exercise, where glycolytic ATP synthesis can be neglected, this results in the alkalinization of the cytosol to a degree that depends on the total buffer capacity of the cytosol β, which can be estimated from the corresponding changes in pH and PCr as β = γδ[PCr]/δpH (which is positive when δpH is positive and δ[PCr] is negative). The non-Pi component of cytosolic buffer capacity (β_NP_) is then calculated by subtracting the calculated buffer capacity due to measured Pi (33). In general, this calculation was performed for the interval from rest to the exercise data point of maximum positive δpH measured from rest.

Postexercise changes in pH and PCr were used to estimate initial-recovery rates of net H^+^ efflux as described in Ref. (36). Briefly, H^+^ efflux *E* is estimated as the sum of the rate at which H^+^ is generated as a consequence of oxidative PCr resynthesis, plus the rate at which H^+^ are released by cytosolic buffers as pH increases back toward basal values (the calculation still works in early recovery, where the release of H^+^ by PCr resynthesis typically exceeds the rate of H^+^ efflux, so that pH initially falls below the end-exercise value). Thus *E* = (βδpH − γδ[PCr])/δ*t* (remember that γ is negative). In practice, the spectrum-to-spectrum cumulative sum of (βδpH − γδ[PCr]) increases during recovery in a way well-fitted by a single exponential, and so such a fit is used here to estimate the true initial-recovery rate *E* for both localized datasets, and for pH1, the nonacid component in the nonlocalized dataset. For pH2, the acid component in the nonlocalized dataset, the lack of apparent recovery of pH in early recovery implies that the cumulative sum (βδpH − γδ[PCr]) goes initially negative, and monoexponential kinetics therefore do not apply. In this case, linear regression is used to quantify the small apparently negative postexercise rate of H^+^ efflux (i.e., net intracellular H^+^ generation). These H^+^ efflux calculations assume the mean value of β_NP_ established from the corresponding initial-exercise calculation described earlier, adding the relevant calculated contribution of Pi.

### Imaging Sequences

Exercise leads to a change in the *T*_2_* in muscle (37, 38). To establish which muscle groups subjects had used, the time courses of values in *T*_2_* maps, one acquired prior to exercise and nine acquired in succession immediately after exercise, were assessed. These imaging data were acquired for one subject in a short session appended to the study's standard spectroscopy protocol. A 2-D multiecho gradient echo sequence was used, with five slices of 4-mm thickness, 0.8-mm gap, a matrix size of 128 × 104 and a field of view of 148 × 120 mm (1.2-mm voxels). Eight echoes were recorded, at *T*_E_ = {4, 9, 14, 19, 24, 29, 34, 39} ms, with no parallel imaging acceleration, in 18 s. There were ∼25 s between multiecho gradient echo scans.

### Processing of Imaging Data

*T*_2_* maps were calculated via a weighted least-squares fit to natural logarithmic voxel values, using Matlab (Mathworks, Inc., Natick, MA). All *T*_2_* maps were coregistered to the *T*_2_* map acquired immediately after exercise using functional MRI of the brain software library (FMRIB) software library (FSL)'s FMRIB's linear image registration tool (FLIRT) (39), by applying parameters derived from coregistering the corresponding gradient echo (GE) images at the first echo time.

## RESULTS

**Fig 3:**
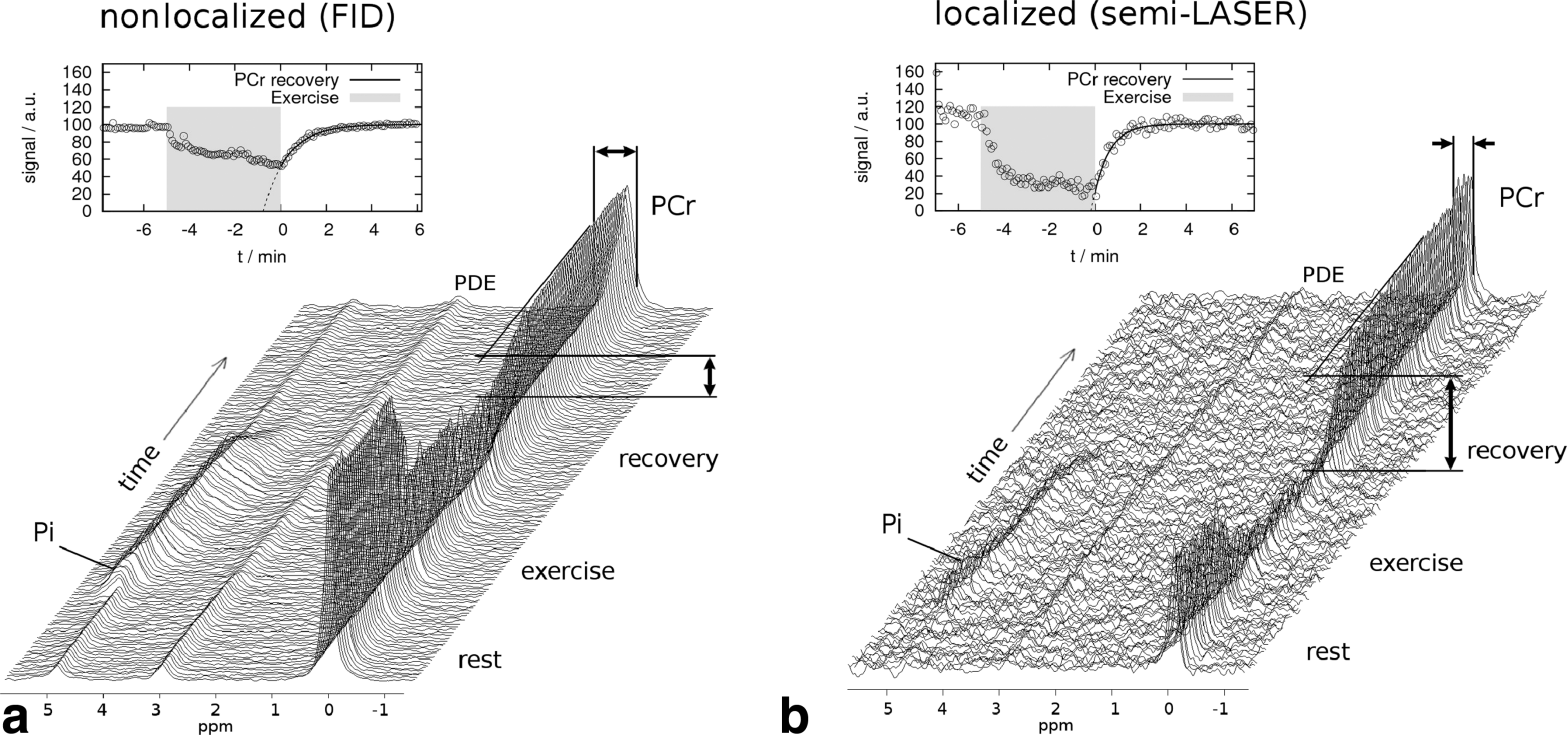
Stack plots of ^31^P spectra of exercising muscle acquired without localization (a) and with semi-LASER localization (b) in a single subject. The insets on top show fitted PCr amplitudes and exponential PCr recovery fits for the respective data sets. Localization to the exercising muscle leads to stronger detected PCr depletion and improved, lower line widths (indicated by arrows). Apodization (30 Hz) was applied to simplify the visual appearance.

Plantar flexion exercise led to depletion of PCr, accompanied by an increase in Pi and a drop in pH in all subjects. Figure 3 shows typical time courses of ^31^P NMR spectra at the region around PCr and Pi, acquired without (a) and with localization (b). Data represent single acquisitions with 6 s time resolution, exercise intensity was 50% MVC. Localized data shown are from the second localized bout, therefore the subject had already performed the exercise and rested before the acquisition of each of the data sets. The insets above the stack plots show the PCr amplitudes of the corresponding data sets, as fitted with AMARES (i.e., peak area in spectrum) and the monoexponential fit to quantify the PCr recovery parameters depletion *d*, resynthesis rate constant *k*, and equilibrium magnetization *m* of the PCr recovery. Data are scaled to *m* = 100 arbitrary units for display. Data shown in Fig. 3 indicate that localization of the signal to an exercising muscle leads to larger measured PCr depletion at the same exercise intensity (indicated by vertical arrows in the stack plots) and improved line widths (horizontal arrows).

### Differences Between Localized and Nonlocalized Data

In nonlocalized experiments (bout 2), PCr fell by 53 ± 10% across subjects at the end of exercise with 50% MVC (*n* = 7). In the localized experiments which followed and were conducted with the same exercise intensity (bout 3), the measured PCr decrease was 79 ± 7%, indicating a significantly (*P* = 0.002) higher sensitivity to PCr depletion in the exercising muscle with localized measurements. These data are representative for *n* = 7 subjects who exercised at a similar intensity of 50% MVC, which lead to PCr depletion of at least 66% in the localized experiments. Two subjects exercised at 30% MVC, which lead to PCr depletion in the localized spectra of 25 and 27%, respectively. One subject was excluded from analysis due to strong motion during both exercise and recovery, evidenced by PCr line width. Statistical significance of the differences between values measured during the second (nonlocalized) and third (localized) bout were tested using the two-tailed paired *t*-tests.

The SNR of resting PCr measured as peak height/SD of noise after application of a Lorentzian matched filter was 43 ± 11 in localized spectra (*T*_R_ = 6 s, no averaging) and 266 ± 65 in nonlocalized spectra. The relative standard deviation of PCr depletion *d* across seven subjects performing the stronger exercise was 25% in the first bout (localized measurement), 22% in the second (nonlocalized), and 9% in the third bout (localized).

### Time Course of PCr Signal Intensity

**Fig 4:**
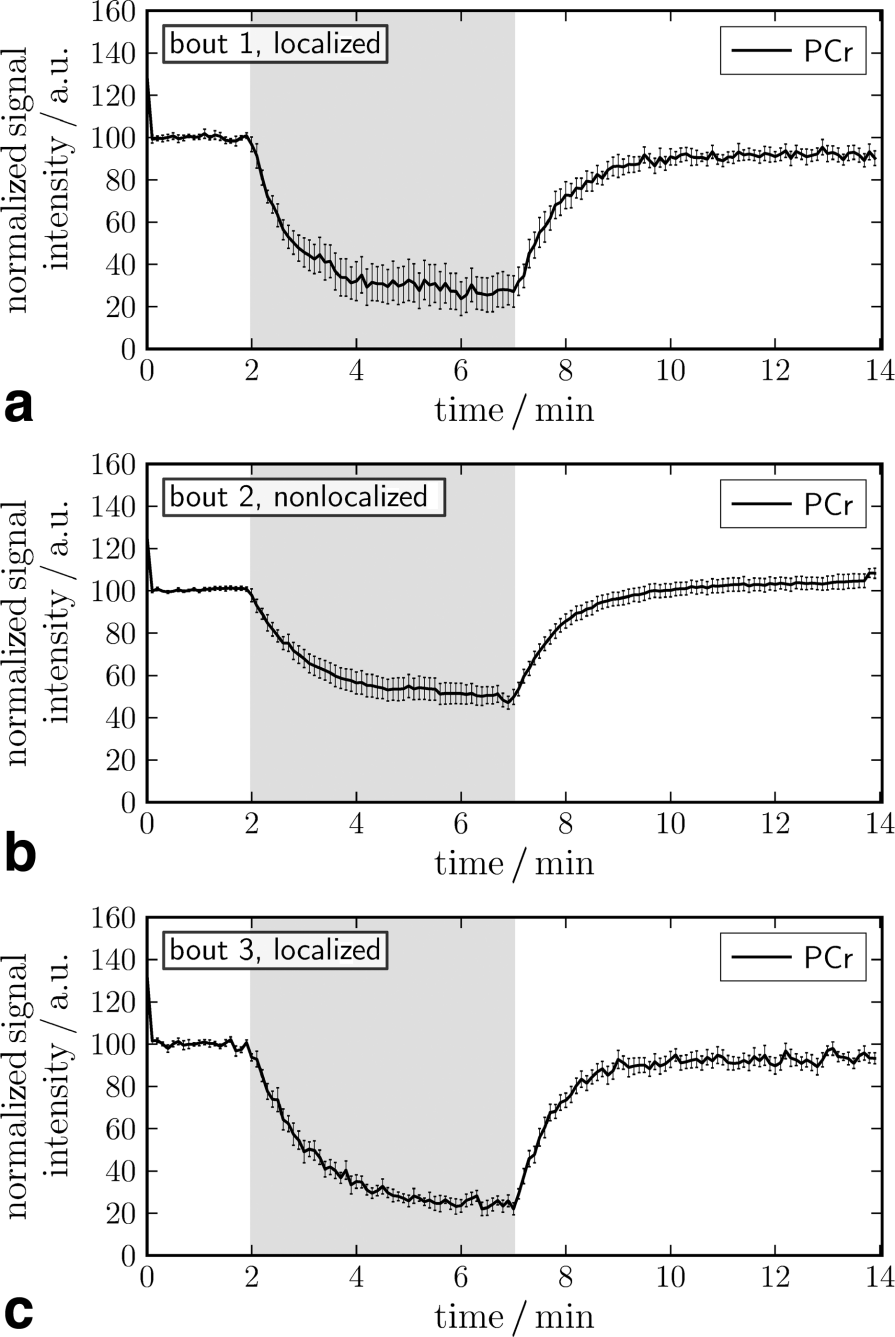
Time courses of PCr signal intensity from three exercise bouts of equal intensity measured with (a), without (b), and with (c) ^31^P MRS localization to gastrocnemius medialis muscle. Data represent mean ± standard error of the mean (SE) over seven subjects exercising at 50% MVC and are normalized to resting PCr signal intensity. The exercise period is indicated in gray.

Time courses of PCr during rest, exercise, and recovery expressed as averages across subjects are shown in Fig. 4.

**Fig 5:**
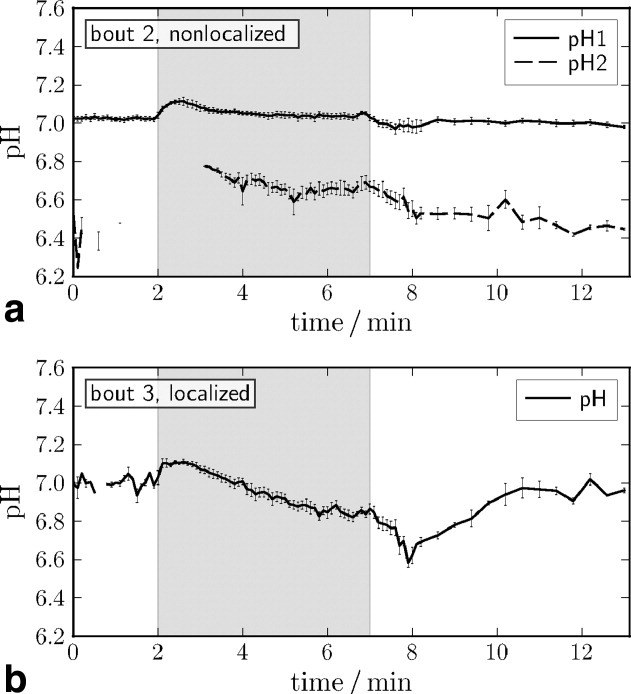
Time course of pH during rest, exercise, and early recovery (mean ± SE). The nonlocalized measurements demonstrated a peak-splitting of the Pi resonance during exercise, which indicates signal contributions by weaker- and nonexercising muscle, other than medial gastrocnemius. This contamination effect was absent in the localized measurements.

**Fig 6:**
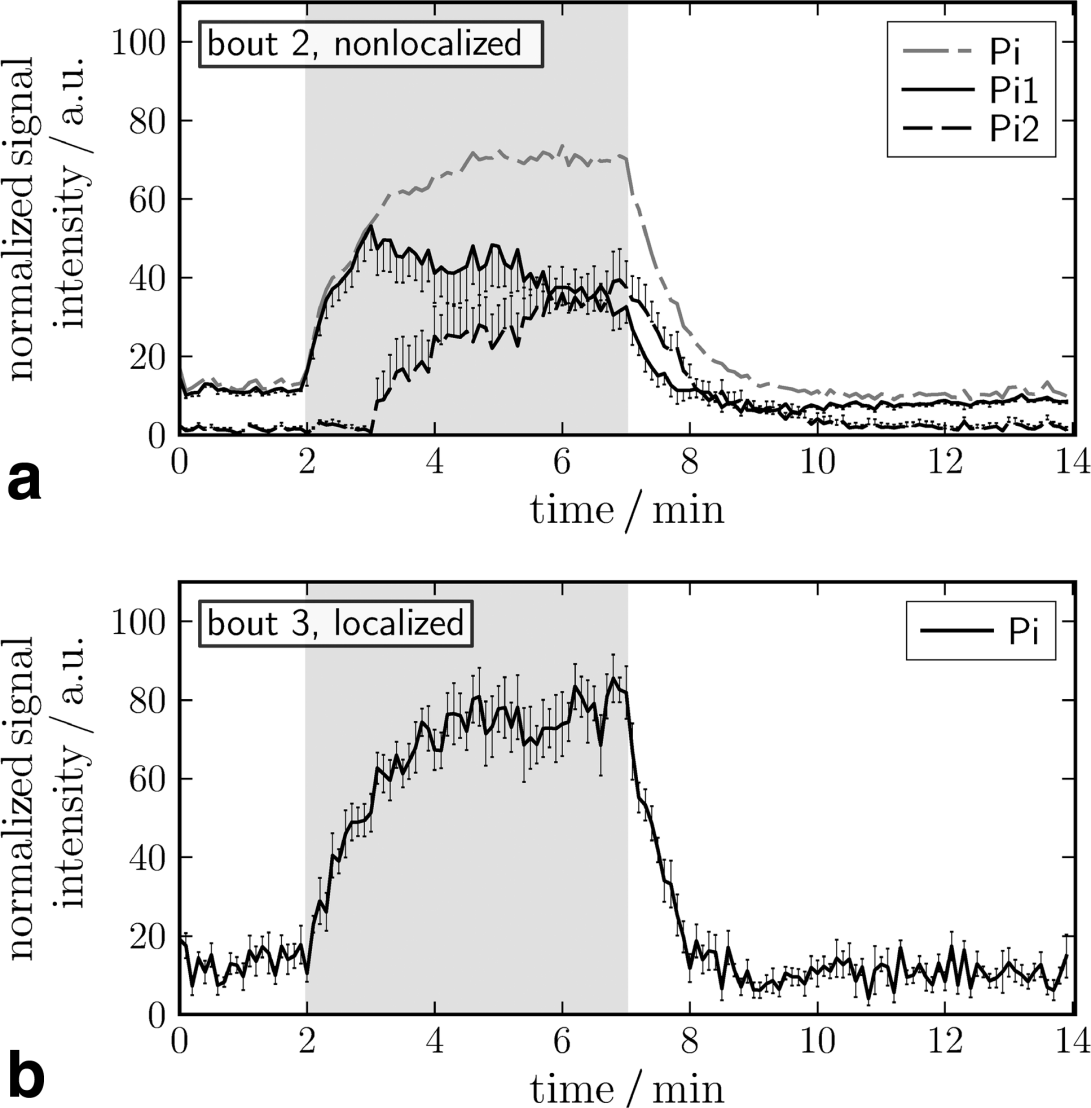
Time course of Pi (mean ± SE). Pi2 is not present during early exercise and becomes discernible after 1–3 min after onset of exercise, in nonlocalized spectra only (see also Fig. 5). After an initial rise, the Pi1 moiety decreases as pH falls, and signal gets attributed to the more acidic Pi2.

**Fig 7:**
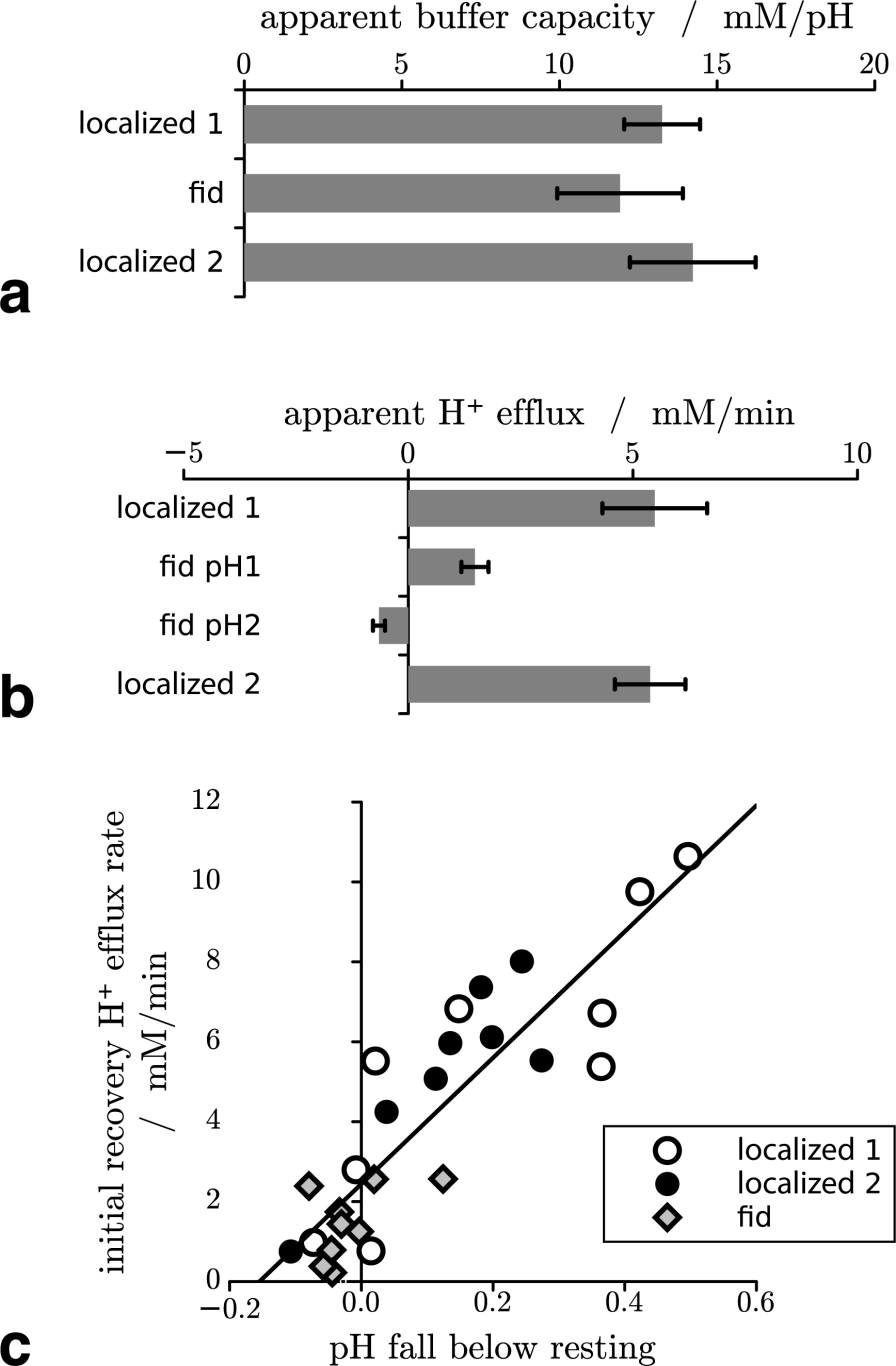
Cytosolic non-Pi buffer capacity (β_NP_) at initial exercise (a) and initial postexercise rates of H^+^ efflux (*E*) calculated at early recovery (b), bars represent mean ± SE in *n* = 9 subjects. Initial recovery H^+^ efflux rate vs. end-exercise pH fall below resting value for individual subjects (c).

**Fig 8:**
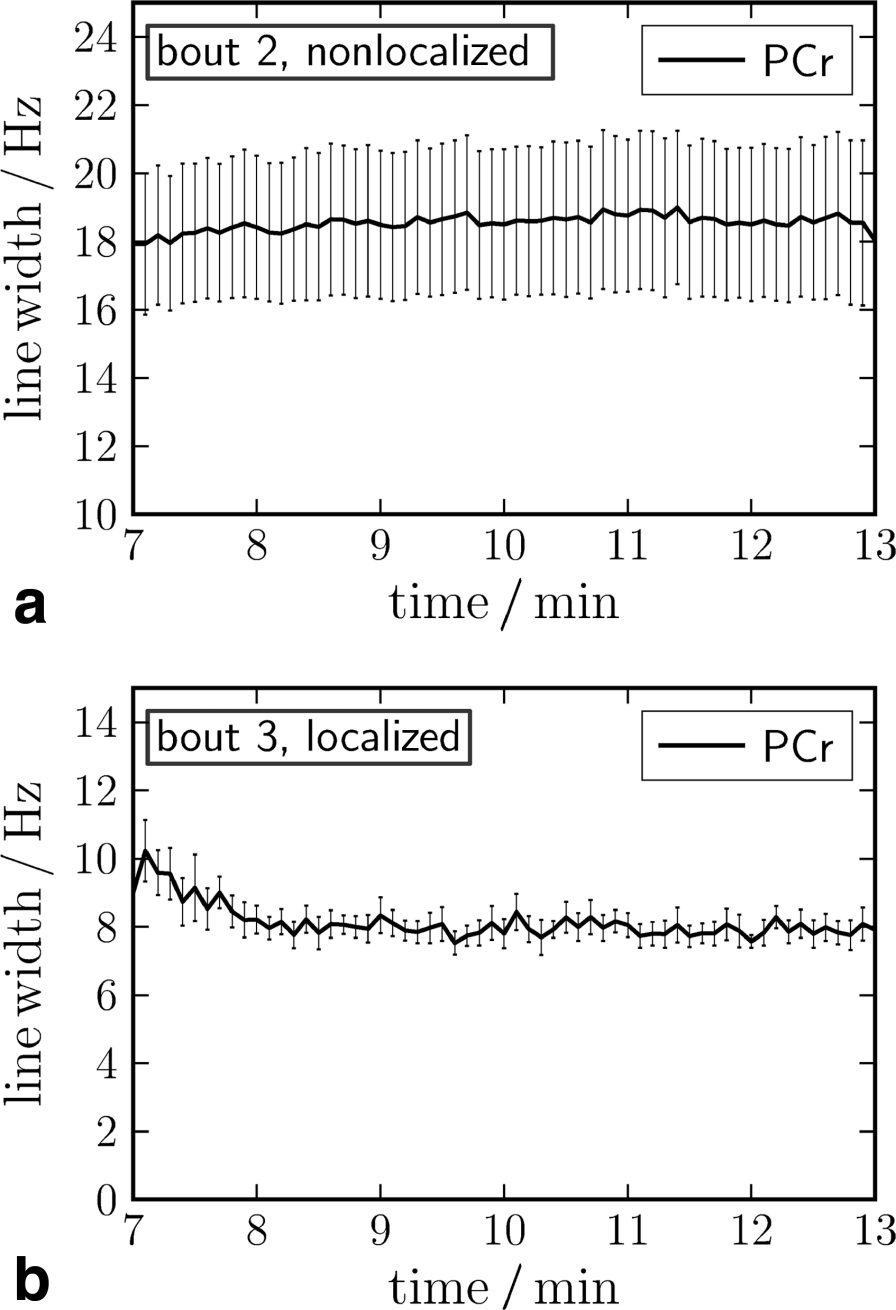
Line width of the PCr resonance measured during recovery from aerobic exercise in seven subjects (mean ± SE). Measurements were performed without/with localization during subsequent exercise bouts.

The top and bottom panel (Fig. 4a,c) show data from gastrocnemius muscle, localized using the semi-LASER sequence. The middle panel (Fig. 4b) shows nonlocalized data. Data in Fig. 4a–c were acquired in consecutive exercise bouts. The primary objective of the first bout was to ensure reproducible exercise conditions in the following bouts. For that reason, Figs. 5–8 show data from the second and third exercise bout only (*n* = 7, similar exercise intensity). In this section, results are compared between the second (nonlocalized measurement) and third bout (localized measurement) or between all three bouts, as indicated. All signal intensities are normalized, so that resting PCr peak intensity is 100.

There was a trend (*P* = 0.13) toward faster PCr recovery with localized measurements compared to nonlocalized measurements (*t*_1/2_ = 34 ± 7 s c.f. *t*_1/2_ = 41 ± 7 s, respectively). The initial PCr resynthesis rate is the slope d[PCr]/d*t* = *k*ċ*d* of the recovery curve at beginning of recovery and *k* is inversely proportional to the recovery time constant. The initial PCr resynthesis rate was found to be elevated to 1.0±0.1 a.u. when using localization, compared to 0.5±0.1 a.u. (nonlocalized) in units of equilibrium signal *m*/s (corresponding to %/s, as *m* = 100). Making the standard assumption that [ATP] = 8.2 mmol/L cell water across the whole muscle, these initial recovery rates correspond to 17 ± 4 (nonlocalized) and 31 ± 5 mmol/l/min (localized), a highly significant difference (*P* = 0.0009).

Results were substantially unchanged, when subjects all subjects were included in the analysis, independent of exercise intensity: measured PCr depletion was significantly higher (*P* = 0.005), recovery half times tended to be shorter (not significant, *P* = 0.31) and initial PCr resynthesis rate was faster (*P* = 0.0016, two-tailed paired *t*-test, *n* = 9) in the localized measurements.

Mean values of PCr depletions, pH changes, PCr recovery half times, and initial PCr resynthesis rates of all subjects (i.e., including stronger and weaker exercising subjects, only excluding data from one subject in which the line width was atypically high) are given in Table 1 for all three exercise bouts. Average data for the first (localized) bout are also given in Table 1. They show the same trends as localized data from the third (localized) bout, and the same differences compared to nonlocalized data, albeit with higher standard deviations and decreased significance levels. Similarly, results from the remaining less strongly exercising subjects also show stronger PCr depletion and faster recovery rates, when localized spectra were acquired.

**Table 1:** PCr Depletions, pH at Rest, end Exercise pH and the Minimum pH Reached Postexercise, PCr Recovery Half Times and Initial PCr Resynthesis Rates as Mean ± SD of All Subjects (Performing Strong and Weaker Exercise) Measured With and Without Localization

Bout	*n*	Relative PCr depletion (%)	Resting pH	End exercise pH	Post exercise pH_min_	PCr recovery *t*_1/2_ / s	d[PCr]/d*t* (mM/min)
Localized 1	7	83 ± 14**	7.01 ± 0.03	6.73 ± 0.18	6.52 ± 0.15	39 ± 9	28 ± 8*
Nonlocalized	7	54 ± 9	7.03 ± 0.04	6.68 ± 0.08	6.47 ± 0.08	42 ± 7	17 ± 4
Localized 2	7	79 ± 7**	7.04 ± 0.03	6.87 ± 0.07	6.63 ± 0.03*	34 ± 7	31 ± 5***
*Weaker exercising subjects*							
Localized 1	3	37 ± 8*	7.05 ± 0.04	7.05 ± 0.03	6.71 ± 0.20	25 ± 1	12 ± 5*
Nonlocalized	2	24 ± 8	7.04 ± 0.01	7.09 ± 0.02	7.02 ± 0.01	31 ± 4	11 ± 4
Localized 2	2	26 ± 1	6.99 ± 0.01	7.07 ± 0.03	6.86 ± 0.10	35 ± 25	14 ± 9

is split in nonlocalized data, pH is given for the more acidic moiety. Significant differences between the localized and the nonlocalized data are indicated as

* *p* < 0.05;

** *p* < 0.01;

*** *p* < 0.001.

### pH and Pi

Intracellular pH, quantified using the chemical shift between Pi and PCr in single subjects' spectra, is plotted in Fig. 5 for all subjects who exercised strongly. Results quantified without temporal averaging of spectra (6 s time resolution) are shown during rest, exercise, and the first minute of recovery. Individual subjects' pH at each respective time point was taken into account in Fig. 5 when the SNR of Pi reached a threshold of 2.6, which was found to result in reliable pH quantification. The remaining data from later recovery, where Pi is too low for pH quantification from nonaveraged spectra, were obtained from spectra averaged in blocks of four, which leads to a lower time resolution of 24 s in the later recovery period (no moving average). Therefore, some points in Fig. 5 may not be representative for the entire group. In particular, points without error bars represent only one subject.

Time courses of the corresponding Pi peak amplitudes are shown in Fig. 6. This resonance was not split in localized data but showed a distinct split in all but one nonlocalized data set, acquired under the same exercise conditions. The pH time course for nonlocalized measurements (Fig. 5a) therefore shows two lines, for a more alkaline Pi1 and a more acidic Pi2, separated by a gap that corresponds to the chemical shift between the Pi peaks when they become discernible. For a quantitative fraction of the two Pi compartments in nonlocalized data see Fig 6a. Localized measurements, with an unsplit Pi resonance, only consist of a single value of pH at each time point. An increase in line width from 15 ± 3 to 49 ± 9 Hz was observed with localized acquisitions in the subjects performing stronger exercise. The line width change was less pronounced in nonlocalized spectra, where Pi1 line width increased from typically 40 Hz at rest and early exercise to 50 Hz, while Pi2 line width was 30 Hz during exercise. As 40 Hz corresponds to 0.33 ppm, it is evident that discrimination of two Pi peaks is only reliable if separation is sufficient, i.e., from ∼0.3 ppm, corresponding to a pH difference of 0.2 units.

All pH curves show an initial phase of alkalinization after onset of aerobic exercise and a drop during the following minutes of exercise. After exercise, the pH continues to decrease to its minimum for 140 ± 90 s. The minimum pH reached postexercise (see Table 1) was measured in spectra from four averaged time points of single subjects in the localized data.

The time course of Pi concentrations in Fig. 6 shows an increase after onset of exercise and the postexercise decrease. From the relative amplitudes of the split Pi peaks in nonlocalized data, the fraction of the more acidic to the more alkaline pH compartments can be estimated. The calculated sum of Pi1 and Pi2 is shown in Fig. 6a with a dash-dotted line. Note that Pi1 of nonlocalized measurements at the onset of the measurements is reflecting increase in total Pi, as pH changes at that time were too small to result in separation of Pi1 and Pi2.

### Cellular H^+^ Buffering and Efflux

The cytosolic non-Pi buffer capacity (β_NP_) calculated from pH and PCr changes during initial exercise (see “Subjects and Methods” section) is shown in Fig. 7a. There is evidence of a difference between neither the two exercise intensities nor the localized and nonlocalized data, and results are broadly consistent with other published data (e.g., Refs.^40^, ^41^).

Fig. 7b shows the initial postexercise rates of H^+^ efflux (*E*), calculated from changes in pH and PCr (see “Subjects and Methods” section). As expected on the basis of the pH-dependence of the various processes contributing to net H^+^ efflux in muscle, higher rates are seen for the localized data, where pH changes during exercise are larger, than for pH1, the nonacidifying pH in the nonlocalized data. As noted in “Subjects and Methods” section, the slow or absent recovery of pH2 in the nonlocalized data imply a negative rate of H^+^ efflux, i.e., a transient cytosolic H^+^ accumulation.

The common pH-dependence of H^+^ efflux in the two localized datasets and the nonlocalized pH1 can be seen in Fig. 7c, which plots individual-subject values of efflux against the end-exercise fall in pH below the basal value.

### PCr Line Width Changes

Time courses of the line width of PCr after 5 min exercise are shown in Fig. 8. Results in Table 2 are averages over consecutive time points of the line widths averaged across subjects, given for rest, early recovery and 5 min after start of recovery. During exercise (not shown), PCr line width in localized spectra varied between 8 and 13 Hz, possibly due to motion. Note that the intrinsic line width of PCr in resting calf muscle at 7 T is 4.7 Hz (*T*_2_ = 217 ± 14 ms; Ref.^23^). Similar to data for metabolite concentrations, pH values and PCr recovery rates, line widths show a higher intersubject variability (error bars in Fig. 8) during the first bout than in following localized measurements. With localized ^31^P MRS, a distinct decrease of PCr line width from an elevated value immediately after exercise toward the resting value is observed (Fig. 8a, Table 2, bout 2). The difference between the postexercise line width and 5-min recovery is negligible in nonlocalized spectra (exercise bout 2), which further show large intersubject variability.

**Table 2:** Line Width of PCr in Hz at Rest (Mean ± SD of 10 Time Points), Immediately After Exercise (3 Points), 5 min Later (10 Points) Δ_recovery_ (The Difference Between the Latter Two Values)

Bout	PCr line width (Hz)			
	Rest	Postexercise	+5 min	Δ_recovery_
1	7.9 ± 0.2	10.4 ± 0.4	8.3 ± 0.1	2.0
2	14.5 ± 0.1	18.0 ± 0.2	18.7 ± 0.2	−0.7
3	8.7 ± 0.2	9.8 ± 0.4	7.9 ± 0.2	1.9
1 + 3	7.9 ± 0.4	10.1 ± 0.5	8.1 ± 0.3	2.0

### Activation Maps

Compared to the pre-exercise baseline, in which *T*_2_*, measured using quantitative *T*_2_* imaging, was ∼21 ms in the gastrocnemius muscle, *T*_2_* was shorter immediately postexercise (∼19 ms) and increased to a maximum (of ∼22 ms) at ∼2-min postexercise.

**Fig 9:**
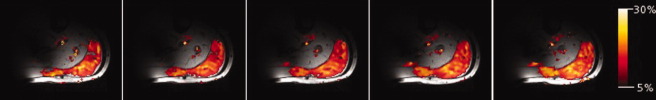
Five consecutive gradient-echo slices of the calf overlaid with percentage change in *T*_2_* between two measurements made immediately postexercise and 2 min subsequently. Exercise-induced changes in *T*_2_* are constrained to the gastrocnemius.

To determine the distribution of exercise-related *T*_2_* changes, the voxel-wise percentage change in *T*_2_* was calculated between that measured immediately postexercise and that measured ∼2 min postexercise. *T*_2_* changes were restricted to, and quite homogeneous within, the gastrocnemius muscle. Voxels in which *T*_2_* increased by more than 5% are illustrated in Fig. 9, overlaid on gradient-echo images at the first echo time. The arbitrary threshold of 5% was applied to reduce noise in the rest of the image. If other muscles were working during the exercise but showed very different response characteristics (e.g., a different time course or opposed pattern of signal change), it is possible that they would not emerge as having been active in this analysis. To test this possibility, MELODIC Independent Component Analysis (42) was applied to the time course of *T*_2_* maps. This identified a single non-noise component, which corresponded to that illustrated in Fig. 9, indicating that no other muscle groups were used.

### DISCUSSION AND CONCLUSION

In this study, we investigated the effect of localizing the acquisition of ^31^P spectra and compare with nonlocalized spectroscopy at the same temporal resolution of 6 s, at rest and aerobic exercise and recovery of human calf muscle. Differences were found between data derived from ^31^P NMR spectra acquired with semi-LASER, a spectroscopic single-shot localization technique and without localization. Measured PCr depletion was greater, and the concomitant initial PCr resynthesis rate was higher in localized data. These differences were highly significant. A trend to shorter PCr recovery time constant was found with localization. Changes in line width were evident in localized data from medial gastrocnemius during recovery, in good agreement with findings from multigradient-echo imaging, which showed exercise-induced *T*_2_ changes that were restricted to this muscle. Further, we found that splitting of the Pi peak during exercise occurs in nonlocalized data only. Estimation of buffer capacity from initial-exercise changes in pH and PCr was apparently reliable by both localized and nonlocalized methods, although estimates of initial-recovery H^+^ efflux by nonlocalized MRS were grossly affected by the Pi splitting during exercise, which gradually disappears during recovery.

The observed pH time course with an initial alkalinization period at onset of exercise and continued acidification during early recovery was expected and is also well known from the literature (43).

The exercise bouts to compare the two acquisition strategies were acquired in one session. As a prior exercise bout potentially alters the parameters measured in the following bouts, an analysis of known possible influences is given here. A greater acidification of the first than following repeated exercises has been reported in humans (44) and mice (45). In the study of Yoshida and Watari (44), a shorter (2 min) and much more strenuous exercise protocol lead to a pH drop to 6.2 units for the more acidic Pi2 moiety during the first bout and was repeated after only 2 min. In all three following bouts, end-exercise pH2 reached similar values of 6.5. Similarly, with repeated submaximal electrical stimulation for 2 min and 10 min rest over 2.5 h, Baligand et al. (45) found the strongest variations in end-exercise pH, force output and PCr recovery time constant between the first and the second bout, after which a steady state was reached. The end exercise pHs of 6.73 and 6.87 we found in bouts 1 and 3 present a relatively small nonsignificant (*P* = 0.2) difference. Rossiter et al. (46) determined the dynamics of intramuscular [PCr] simultaneously with those of pulmonary oxygen uptake (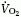) in humans who performed two consecutive bouts of high-intensity knee-extensor exercise 6-min with a 6 min rest interval. Along with changes in 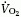, they report a 16% reduction in PCr depletion and no alteration of initial PCr recovery rate. The reduction of PCr depletion by 4% between bouts 1 and 3 reported here is comparably small and statistically nonsignificant (*P* = 0.6). Forbes et al. (47) studied the effect of recovery time during repeated bouts of exercise and found that the influence of previous exercise on intracellular [H^+^] became undetectable after 15 min of rest. Muscle lactate, possibly the driving force of water shifts occurring after exercise, can be measured by ^1^H MRS (48, 49) and decays with similar time constant, on the order of 20 min or less.

That aside, we used the second (nonlocalized) and the third (localized) bout for direct comparison of acquisition strategies, which—in the light of the above-mentioned literature—should suffer from only marginal, if any, preconditioning effect, because the effects of repeating exercises rapidly reach a steady state. Moreover, comparing the first with the third exercise bout (both acquired with localization) reveals no statistically significant differences between derived metabolic parameters. The most notable effect is the lower intersubject variability during later exercise bouts, which appears rather as an advantage of performing repeated exercise with sufficient temporal spacing in one session, as compared to acquisitions on several study days. Layec et al. (50) studied the long-term reproducibility of the assessment of muscle energetics data by ^31^P MRS. To give an exemplary quantitative comparison, the mean coefficients of variance for initial PCr recovery rate, PCr depletion and pH at rest and end of exercise were 35.7, 21, 0.3, and 1.5%, respectively, in the long-term comparison, while the variances between first and third bout in this study were 17, 15, 0.3 and 1.7% for the corresponding quantities. In our interpretation, the high robustness of quantified parameters, i.e., negligible influence of repeating the exercise protocol within one session is the consequence of a metabolically less challenging exercise protocol compared to previous studies (nonlocalized end-exercise pH2 was 6.68) and of a considerably longer interstimulus interval of ca. 30 min.

On first sight, our findings appear to contrast with a recent study reporting no differences between the PCr time courses from pulse-acquire MRS and from ^31^P RARE imaging acquired after the end of forearm exercise in a single subject (18). While that study and ours used similar geometries (single loop coils, with 10.5-cm coil for calf and a 7-cm coil for forearm), there is a fundamental difference in the activation pattern: plantar flexion exercise activates medial gastrocnemius, a superficial muscle covering ca. 20% of the cross-sectional area of the organ, while squeezing a rubber block led to PCr depletion in ca. 80% of the forearm cross section, leaving only a small area opposite the coil nonactivated. Whether pulse-acquire ^31^P MRS yields data which is representative of the activated tissue or not is therefore a question of study design (which extremity is studied, under which stimulus, activating certain muscles) and of available hardware (size and geometry of the ^31^P RF coil).

Because the nonlocalized PCr signal from calf muscle acquired with a 10-cm surface coil during plantar flexion exercise contains contributions from muscles exercising to a highly different extent, the normalized PCr signal depletion is larger in spectra that are localized to exercising muscle only. Using initial slope d[PCr]/d*t* is therefore problematic when applied on data acquired without localization. When comparing two groups, one thereby implicitly assumes that the volume of the exercising muscle over the total muscle volume in the *B*_1_-field of the coil (and by the principle of reciprocity the sensitive volume) is equal in all subjects. Muscle volume may differ between subjects, e.g., as an effect of specific training or diseases. When the coil is placed as close as possible to the exercising muscle, the *B*_1_ field will detect a relatively large PCr signal intensity originating from the exercising muscle, if this particular muscle is large. As a result, PCr depletion will be larger for these subjects, and with it, initial d[PCr]/d*t*. A similar, opposite problem is likely to arise in comparing two groups in which the subjects in one of the groups have more subcutaneous fat. The coil might be expected to detect less nonexercising muscle in this case. Localized spectroscopy avoids this problem.

A single loop coil with a diameter of 10 cm has a sensitive volume of roughly a half sphere with the same radius as the coil, thus the VOI for nonlocalized acquisitions is ca. 300 cm^3^. Receive sensitivity decreases with distance from the coil plane, and with a nonadiabatic pulse-acquire scheme, signal from tissue far from the RF coil plane also does not experience a 90° excitation pulse, when RF is calibrated by maximizing total signal. The gastrocnemius and soleus muscles are those which primarily contribute to nonlocalized MRS, as they are superficial or relatively close to the coil, respectively. Musculus peroneus brevis and m. tibialis posterior and the anterior muscle groups are smaller and located at a distance of more than 5 cm from the coil. Their contribution, even to nonlocalized MR spectra, was considered to be negligible. Not exciting NMR signal in nonsuperficial muscles may be considered an advantage of using nonadiabatic excitation in this case. Localization results in a decrease of the average volume of tissue contributing to the spectral data acquisition to 38 cm^3^. As a consequence of the eightfold smaller VOI, lower SNR is expected for localized measurements (which additionally uses an echo time, and hence results in *T*_2_ decay). In our experiments, the SNR of resting PCr was reduced by only a factor of 4.5 with localized spectroscopy compared to FID acquisitions, comparing SNR calculated without any apodization. Despite the *T*_2_ losses with localization, this can be explained by a threefold narrower line width and a more homogeneous excitation of the VOI due to its smaller size and the adiabatic refocusing.

To limit the sensitive volume to exercising tissue, small surface coils can be used. This is effective for sufficiently large muscles and if activation within the sensitive volume is spatially homogeneous. However, when the activated muscle covers only a fraction of the coil's sensitive volume, the condition to acquire signal only from active tissue is not met. Further, the *B*_1_ and receive sensitivity will be heterogeneous in the small sensitive volume, and the approach of localizing the spectroscopic acquisition with a small surface coil fails, if the exercising muscle is not near the surface.

While Pi line width is known to change due to intracellular pH changes and has been attributed to compartmentation (15), to our knowledge this is the first observation of an exercise-induced line width increase of intramyocellular PCr. Motion seems an unlikely explanation, as the line width decreases slowly on cessation of exercise. The observed effect may be related to myoglobin deoxygenation, as we observe an initial line width increase which is over-compensated for later, during recovery. This line width behavior is consistent with *T*_2_* imaging data acquired for one subject. The effect is far less pronounced in nonlocalized spectra which contain larger fractions of resting tissue and have a larger line width due to stronger *B*_0_ inhomogeneities across the large sensitive volume. Localized spectroscopy appears to be less motion sensitive (cf. pre-exercise and postexercise line widths in Table 2) and shows a lower intersubject variability (smaller error bars in Fig. 8) than nonlocalized experiments, which makes it possible to detect line width changes of PCr.

## SUMMARY

This study demonstrates that localized acquisition of dynamic ^31^P MRS data during a calf muscle exercise protocol yields results that differ significantly from results achieved with widely used nonlocalized MRS acquisitions. The application of a relatively simple and very robust localization method is demonstrated at 7 T. The same approach can be applied at lower fields. As implemented here, localized dynamic ^31^P MRS yields excellent SNR for PCr with 6 s time resolution and allows accurate quantification of Pi and pH during the entire time course of exercise and recovery. Localized data are more specific to the tissue under investigation and should therefore allow more accurate interpretation of studies in metabolic research and routine investigations of muscle function based on the MRS of high-energy phosphates.
